# A dedicated fungal culture medium is useful in the diagnosis of fungemia: a retrospective cross-sectional study

**DOI:** 10.1371/journal.pone.0164668

**Published:** 2016-10-13

**Authors:** Shuwei Zheng, Tong Yong Ng, Huihua Li, Ai Ling Tan, Thuan Tong Tan, Ban Hock Tan

**Affiliations:** 1 Department Infectious Diseases, Singapore General Hospital, Singapore, Singapore; 2 Department of Pathology, Singapore General Hospital, Singapore, Singapore; 3 Health Services Research & Biostatistics Unit, Division of Research, Singapore General Hospital, Singapore, Singapore; University of Hong Kong, HONG KONG

## Abstract

**Background:**

Mortality for candidemia ranges from 15% to 35%. Current guidelines recommend inoculating blood into three aerobic and three anaerobic blood culture bottles when candidemia is suspected, without mention of a fungal blood culture bottle.

**Objective:**

To determine the value of the BACTEC Myco/F Lytic blood culture media in the diagnosis of fungemia.

**Methods:**

A two-year retrospective cross-sectional study was performed for patients who had fungemia with submitted BACTEC Plus Aerobic/F (Aer), BACTEC Plus Anaerobic/F (Anaer) or Myco/F Lytic (Myco) blood culture bottles.

**Results:**

The detection rate of fungemia was 77.4% in 93 patients with contemporaneously submitted blood culture bottles when limited to only Aer/Anaer culture results. The detection rate improved significantly with the addition of the Myco culture bottle results (p<0.0001). A logistic regression model showed that Myco culture bottle submissions were less useful for patients with appropriate anti-fungal therapy administered within 48 hours [OR = 0.18, 95% CI = (0.06, 0.49), p = 0.001] and those with fungal growth detected within 48 hours [OR = 0.33, 95% CI = (0.12, 0.89), p = 0.001]. Among a subset of patients with concordant blood culture results, those with Myco culture bottles submission allowed earlier fungal detection and speciation by at least one day in 27.5% and 25.0% of the cases respectively.

**Conclusion:**

Our study highlights the importance of a dedicated fungal blood culture when fungemia is clinically suspected. Nearly a quarter of fungemias may be missed if a fungal blood culture is not performed.

## Introduction

The detection of organisms in blood cultures has tremendous clinical significance. Candidemia, in particular, carries a considerable mortality. In one study, mortality ranged from about 15% in those who received anti-fungal therapy within 12 hours of clinical suspicion to about 35% in those who received anti-fungal therapy after 48 hours [1].

Blood cultures are crucial for the detection of candidemia, yet they are only 50% sensitive for candidemia [2]. In its 2012 guidelines, the European Society of Clinical Microbiology and Infectious Diseases recommended that 60ml of blood be sent for culture whenever candidemia was suspected [2]. This 60ml should be inoculated, divided in equal parts, into three sets of aerobic and anaerobic blood culture bottles. The addition of a dedicated fungal blood culture bottle was not mentioned, although the same authors pointed out that *Candida glabrata* was known to be better recovered from fungal blood culture bottles [2].

In an early study, investigators using an isolator system noted that dedicated fungal blood culture submissions yielded results that had an impact on therapy initiation in only a few cases [3]. In this study of 5196 fungal blood culture bottles, 5 patient episodes had therapy decision changes attributable to the fungal bottle submissions. While the authors concluded that the use of fungal culture bottles was not cost-effective, they did not compute the cost of extended stay and poor outcomes with a missed diagnosis.

Vetter et al. compared the performance of BACTEC Plus Aerobic/F (Aer) culture bottles against BACTEC Myco/F Lytic (Myco) culture bottles and the isolator system for the isolation of fungi [4]. They noted that the Aer culture bottle missed several isolates of Cryptococcus and Histoplasma. The isolator system appeared to be better than Myco culture bottles, and even recovered bacterial isolates not grown in BACTEC culture bottles. However, time to positivity for fungal growth was also longer with the isolator system [4].

Kirby et al. also reviewed their hospital's use of Myco culture bottles. The analysis was limited to blood cultures submitted contemporaneously using Aer, BACTEC Plus Anaerobic/F (Anaer) as well as Myco culture bottles. A small number of candidemias was detected only in the Myco culture bottles. The time to detection of *Candida glabrata*, was also shorter with Myco culture bottles. All in all, the authors felt that Myco culture bottles were best reserved for the detection of dimorphic fungi and mycobacteria [5].

These data suggest that the additional yield of a dedicated fungal culture bottle is likely to be small. Nevertheless, failure to detect fungemia has serious consequences, and the increasing empiric use of anti-fungal therapy, which is expensive, has led to calls for better anti-fungal stewardship [6]. The specific role of a dedicated fungal culture bottle has not been studied in an Asian setting, except for a small study using seeded blood culture bottles [7].

In this study, we sought to clarify the additional value of fungal blood culture bottles and the degree of concordance between fungal blood culture and standard aerobic/anaerobic blood culture results. We also wanted to shed light on the patient characteristics that would predict a positive result from fungal culture bottles, thereby potentially enabling early and targeted empiric therapy.

## Materials and Methods

### Setting

A list of patients with positive fungal results on blood cultures received from 1^st^ July 2012 to 30^th^ June 2014 was obtained from the Microbiology Laboratory, Department of Pathology, Singapore General Hospital (SGH). SGH, a 1600-bed acute tertiary hospital, is the largest hospital in the country, offering a wide variety of clinical services including solid organ and hematopoietic stem cell transplant services.

In our hospital, all submitted blood culture bottles were processed and worked up in a microbiology laboratory accredited by the College of American Pathologists. The automated blood culture system used was the BACTEC 9240 series. The culture bottles used were the Aer and the Anaer culture bottles [8], both of which were incubated for 5 days. The dedicated fungal culture bottle was the Myco culture bottle [9], which was incubated for 14 days in accordance with our laboratory’s protocol.

The automated blood culture system analyses the rate and the quantity of carbon dioxide production in the bottles and prompts when positive microbial growth is detected. Once growth is detected from a culture bottle, a Gram stain is performed on the broth of each positively flagged blood culture bottle. This allows the identification of Gram-positive and Gram-negative bacteria, yeasts and mycobacteria. The results determine further subcultures on various agars. Suspicious Gram-positive bacteria are subjected to an acid-fast stain to rule out mycobacteria.

Yeast colonies isolated are sub-cultured onto cornmeal agar and undergo urea hydrolysis testing. The workup of suspected *Cryptococcus* cases includes a CGB (canavanine, glycine, bromothymol blue) agar. All colonies are confirmed with API 20 C AUX (bioMérieux, France) or Bruker MALDI Biotyper (Bruker Daltonik GmbH, Bremen, Germany). As part of laboratory protocol, amplification and sequencing of the internal transcribed spacer (ITS1 and ITS4) are performed when the above methods fail to give a clinically adequate identification.

The results are uploaded onto the patient’s electronic record for clinical action.

### Participants

Patients with a positive fungal culture from any of the Aer, Anaer, and Myco culture bottles had their medical records reviewed for relevant clinical details. Only contemporaneously submitted blood culture sets were eligible for inclusion.

The terms used are defined in Table 1.

**Table 1:** Terminologies used.

Terms	Definition
Contemporaneous blood culture set	Any combination consisting of at least one Aer, one Anaer, and one Myco culture bottle, all of which are collected within 2 hours of one another
Positive contemporaneous blood culture set	A contemporaneous blood culture set with fungal growth detected from any of its culture bottles
Concordant blood culture set	A positive contemporaneous blood culture set with growth from its Myco culture bottle and at least one of its Aer or Anaer culture bottles
“Useful” Myco bottle set	A contemporaneous blood culture set with fungal growth detected **ONLY** from its Myco culture bottle **BUT** not from its Aer/Anaer culture bottles
“Less useful” Myco bottle set	A contemporaneous blood culture set with fungal growth detected from **EITHER** of its Aer or Anaer culture bottle, **INDEPENDENT** of fungal detection from its Myco culture bottle

### Statistical Analysis

The additional yield of a Myco culture bottle was determined in two ways.

In the first analysis (Analysis A), the number of blood cultures that were positive only from the Myco culture bottle (“useful” Myco bottle sets) was determined, and this was expressed as a fraction of all positive contemporaneous blood culture sets. In this analysis, a positive contemporaneous blood culture set was counted as one positive isolate and would contribute to the denominator, regardless of previous identical isolates from the same patient. As a subgroup analysis (Analysis A-1), we looked at the effects of microbiological and clinical factors that could be associated with “useful” Myco bottle sets and “less useful” Myco bottle sets. This is done by means of univariate Generalised Linear Latent and Mixed Model.

In the second analysis (Analysis B), we only analysed the first positive contemporaneous blood culture set from a given patient as a unique patient episode and omitted subsequent positive contemporaneous blood culture sets from the same patient. For patients with isolation of multiple fungi in their blood cultures, we also analysed only their first positive contemporaneous blood culture. Using this analysis, the detection rate of fungemia using only Aer/Anaer culture bottles and using both Aer/Anaer and Myco culture bottles was compared using the McNemar’s test [10] at a significance level of 0.05. As a subgroup analysis (Analysis B-1), we again looked at the effects of microbiological and clinical factors that could be associated with “useful” Myco bottle sets and “less useful” Myco bottle sets. This is evaluated by means of logistic regression.

Both subgroup analyses (Analysis A-1 and B-1) were done using R 3.1.3 [11] and STATA 13 [12]. A p value of <0.05 was considered statistically significant.

Finally, among the concordant blood culture sets, we compared the time to fungal positivity and the time to speciation of the Aer/Anaer culture bottles versus the Myco culture bottles (Analysis C).

The study was approved by the SingHealth Centralised Institutional Review Board. An application for waiver of informed consent was granted by the institutional review board as this was an observational study.

## Results

Between 1^st^ July 2012 and 30^th^ June 2014, a total of 83,963 Aer, 83,083 Anaer, and 7,921 Myco culture bottles were processed by the microbiology laboratory of our hospital. A total of 350 blood culture bottles were positive for fungi from 160 patients. Out of these, 234 culture bottles came from 144 contemporaneous blood culture sets from 93 patients.

### Analysis A

A total of 144 positive contemporaneous blood culture sets were positive for fungi (Table 2). Of these, 35 sets (24.3%) were “useful” Myco bottle sets and 109 sets (75.7%) were “less useful” Myco bottle sets. Of the “less useful” Myco bottle sets, 57 sets were positive from both the Aer/Anaer and Myco culture bottles (concordant blood culture sets), and 52 sets were positive from only the Aer/Anaer culture bottles. Therefore, according to Analysis A, the additional yield of the Myco culture bottle was 24.3%.

**Table 2:** Types of fungi cultured from contemporaneously submitted blood culture sets.

*Fungal species*	No. of sets, n = 144 (%)	“Useful” Myco Sets	“Less useful” Myco Sets	
		No. of sets + from Myco only, n = 35 (%)	No. of sets + from Aer/Anaer & Myco, n = 57 (%)	No. of sets + from Aer/Anaer only, n = 52 (%)
*C*. *tropicalis*	44 (30.6)	6 (17.1)	21 (36.8)	17 (32.7)
*C*. *glabrata*	37 (25.7)	12 (34.3)	11 (19.3)	14 (26.9)
*C*. *albicans*	31 (21.5)	8 (22.9)	9 (15.8)	14 (26.9)
*C*. *parapsilosis*	17 (11.8)	7 (20.2)	8 (14.0)	2 (3.8)
*T*. *asahii*	8 (5.6)	0 (0)	5 (8.8)	3 (5.8)
*C*. *dubliniensis*	2 (1.4)	0 (0)	1 (1.8)	1 (1.9)
*C*. *neoformans*	2 (1.4)	0 (0)	1 (1.8)	1 (1.9)
*Candida sp*. *(non-albicans)*	1 (0.7)	1 (2.9)	0 (0)	0 (0)
*S*. *cervisiae*	1 (0.7)	0 (0)	1 (1.8)	0 (0)
*Ustilago species*	1 (0.7)	1 (2.9)	0 (0)	0 (0)

### Analysis B

By patient episode analysis (Analysis B), a total of 93 patients had contemporaneous blood culture sets. Out of these, 21 patients had their first positive contemporaneous blood culture set deemed as “useful” Myco bottle sets. The detection rate was 77.4% when using only Aer/Anaer culture bottles, and increased to 100% with the addition of the Myco culture bottles. McNemar’s test showed that this increase in detection rate was statistically significant (p<0.0001).

Thus, both analyses, whether by all positive contemporaneous blood culture sets (Analysis A) or by patient episodes (Analysis B), demonstrated that not sending Myco bottles would have missed about a quarter of fungemias.

### Analysis A-1

For Analysis A-1, we explored clinical and microbiological factors associated with “useful” Myco bottle sets and “less useful” Myco bottle sets. The only statistically significant factor was that an administration of the appropriate anti-fungal therapy within 48 hours is associated with a “less useful” Myco culture set (i.e. the Aer/Anaer culture results would have been positive regardless of Myco culture result positivity). [OR = 0.25, 95% CI = (0.11, 0.57), p = 0.001] (Table 3).

**Table 3:** The effects of potential factors in predicting the detection of fungal infection using fungal culture bottle by means of univariate Generalised Linear Latent and Mixed Model (Analysis A-1).

Patient Characteristic	No. of Myco culture bottles (%)		OR (95% CI)	P value^ƚ^
	“Less useful”	“Useful”		
Growth of candida				
• No	11 (10.1)	1 (2.9)	Reference	
• Yes	98 (89.9)	34 (97.1)	3.82 (0.47, 30.67)	0.208
Candida species: *C*. *albicans*				
• No	86 (78.9)	27 (77.1)	Reference	
• Yes	23 (21.1)	8 (22.9)	1.11 (0.44, 2.76)	0.826
Candida species: *C*. *tropicalis*				
• No	71 (65.1)	29 (82.9)	Reference	
• Yes	38 (34.9)	6 (17.1)	0.39 (0.15, 1.01)	0.053
Candida species: *C*. *glabrata*				
• No	84 (77.1)	23 (65.7)	Reference	
• Yes	25 (22.9)	12 (34.3)	1.75 (0.77, 4.01)	0.184
Candida species: *C*. *parapsilosis*				
• No	99 (90.8)	28 (80.0)	Reference	
• Yes	10 (9.2)	7 (20.0)	2.47 (0.86, 7.09)	0.092
ICU stay				
• No	53 (48.6)	14 (40.0)	Reference	
• Yes	56 (51.4)	21 (60.0)	1.42 (0.65, 3.08)	0.375
Ongoing TPN use				
• No	93 (85.3)	30 (85.7)	Reference	
• Yes	16 (14.7)	5 (14.3)	0.97 (0.33, 2.87)	0.954
Presence of central line				
• No	19 (17.4)	6 (17.1)	Reference	
• Yes	90 (82.6)	29 (82.9)	1.02 (0.37, 2.80)	0.969
Dialysis within last 3 days				
• No	69 (63.3)	24 (68.6)	Reference	
• Yes	40 (36.7)	11 (31.4)	0.79 (0.35, 1.78)	0.571
Broad spectrum antibiotics within last 1 week^ǂ^				
• No	8 (7.3)	1 (2.9)	Reference	
• Yes	101 (92.7)	34 (97.1)	2.69 (0.32, 22.32)	0.359
Abdominal surgery within last 1 week				
• No	98 (89.9)	31 (88.6)	Reference	
• Yes	11 (10.1)	4 (11.4)	1.15 (0.34, 3.87)	0.822
Ongoing pancreatitis				
• No	100 (91.7)	32 (91.4)	Reference	
• Yes	9 (8.3)	3 (8.6)	1.04 (0.27, 4.08)	0.953
Neutropenia (ANC<500/μL)				
• No	89 (81.7)	31 (88.6)	Reference	
• Yes	20 (18.3)	4 (11.4)	0.57 (0.18, 1.81)	0.344
Prolonged neutropenia >10 days				
• No	94 (86.2)	34 (97.1)	Reference	
• Yes	15 (13.8)	1 (2.9)	0.18 (0.02, 1.45)	0.108
Transplant recipient				
• No	95 (87.2)	30 (85.7)	Reference	
• Yes	14 (12.8)	5 (14.3)	1.13 (0.38, 3.40)	0.827
Transplant type: allogenic HSCT				
• No	107 (98.2)	35 (100)	Reference	
• Yes	2 (1.8)	0 (0)	-	-
Transplant type: liver transplant				
• No	103 (94.5)	32 (91.4)	Reference	
• Yes	6 (5.5)	3 (8.6)	1.61 (0.38, 6.80)	0.518
Transplant type: heart or lung transplant				
• No	106 (97.2)	34 (97.1)	Reference	
• Yes	3 (2.8)	1 (2.9)	1.04 (0.10, 10.32)	0.974
Transplant type: renal transplant				
• No	106 (97.2)	34 (97.1)	Reference	
• Yes	3 (2.8)	1 (2.9)	1.04 (0.10, 10.32)	0.974
Received prolonged at least moderate dose steroids^§^				
• No	100 (91.7)	30 (85.7)	Reference	
• Yes	9 (8.3)	5 (14.3)	1.85 (0.58, 5.95)	0.301
Use of T cell suppression within 90 days^¥^				
• No	74 (67.9)	24 (68.6)	Reference	
• Yes	35 (32.1)	11 (31.4)	0.97 (0.43, 2.20)	0.940
Underlying HIV				
• No	107 (98.2)	35 (100)	Reference	
• Yes	2 (1.8)	0 (0)	-	-
Underlying malignancy				
• No	64 (58.7)	21 (60.0)	Reference	
• Yes	45 (41.3)	14 (40.0)	0.95 (0.44, 2.06)	0.893
Malignancy type: solid organ malignancy				
• No	85 (78.7)	25 (73.5)	Reference	
• Yes	23 (21.3)	9 (26.5)	1.33 (0.55, 3.24)	0.530
Malignancy type: hematological malignancy				
• No	87 (79.8)	30 (85.7)	Reference	
• Yes	22 (20.2)	5 (14.3)	0.66 (0.23, 1.89)	0.439
Underlying chronic liver disease				
• No	95 (87.2)	32 (91.4)	Reference	
• Yes	14 (12.8)	3 (8.6)	0.64 (0.17, 2.36)	0.498
Underlying autoimmune disease				
• No	104 (95.4)	33 (94.3)	Reference	
• Yes	5 (4.6)	2 (5.7)	1.26 (0.23, 6.80)	0.788
Use of azole within 1 week				
• No	89 (81.7)	28 (80.0)	Reference	
• Yes	20 (18.3)	7 (20.0)	1.11 (0.43, 2.90)	0.828
Use of echinocandin within 1 week				
• No	89 (81.7)	27 (77.1)	Reference	
• Yes	20 (18.3)	8 (22.9)	1.32 (0.52, 3.33)	0.558
Fulfils MSG rule [13]				
• No	96 (88.1)	34 (97.1)	Reference	
• Yes	13 (11.9)	1 (2.9)	0.22 (0.03, 1.72)	0.148
Fungal growth detection within 48h				
• No	38 (34.9)	19 (54.3)	Reference	
• Yes	75 (76.1)	16 (45.7)	0.45 (0.19, 1.08)	0.074
Administration of appropriate anti-fungal therapy within 12 hours				
• No	43 (39.4)	16 (45.7)	Reference	
• Yes	66 (60.6)	19 (54.3)	0.77 (0.36, 1.67)	0.513
Administration of appropriate anti-fungal therapy within 24 hours				
• No	34 (31.2)	16 (45.7)	Reference	
• Yes	75 (68.8)	19 (54.3)	0.54 (0.25, 1.17)	0.119
Administration of appropriate anti-fungal therapy within 48 hours				
• No	17 (15.6)	15 (42.9)	Reference	
• Yes	92 (84.4)	20 (57.1)	**0.25 (0.11, 0.57)**	**0.001**

Abbreviations: ICU = intensive care unit; TPN = total parenteral nutrition; ANC = absolute neutrophil count; HSCT = hematopoietic stem cell transplant; HIV = human immunodeficiency virus.

ƚRefer to the Methods section for the calculation of p values

ǂBroad-spectrum antibiotics include 3rd, 4th, and 5th generation cephalosporins, amoxicillin-clavulanate, anti-pseudomonal penicillins, carbapenems, aminoglycosides, fluoroquinolones, aztreonam, tigecycline

§Mean minimum dose of 0.3 mg/kg/day of prednisolone equivalent for 13 weeks

¥Cyclosporine, TNF-α blockers, specific monoclonal antibodies, or nucleoside analogues

### Analysis B-1

With Analysis B-1, we looked at the same factors as in Analysis A-1 but using first patient-episodes. Again, the statistically significant factor was that an administration of appropriate anti-fungal therapy within 48 hours is associated with a “less useful” Myco bottle set [OR = 0.18, 95% CI = (0.06, 0.49), p = 0.001]. In addition, patients with fungal growth detected within 48 hours are associated with a “less useful” Myco culture set (i.e. the Aer/Anaer culture results would have been positive regardless of Myco culture result positivity). [OR = 0.33, 95% CI = (0.12, 0.89), p = 0.023] (Table 4).

**Table 4:** The effects of potential factors in predicting the detection of fungal infection using fungal culture bottles by means of univariate logistic regression (Analysis B-1).

Patient Characteristic	No. of Myco culture bottles (%)		OR (95% CI)	P value^ƚ^
	“Less useful”	“Useful”		
Growth of candida				
• No	6 (8.3)	0 (0)	Reference	
• Yes	66 (91.7)	21 (100)	-	-
Candida species: *C*. *albicans*				
• No	56 (77.8)	13 (61.9)	Reference	
• Yes	16 (23.2)	8 (38.1)	2.15 (0.74, 6.08)	0.149
Candida species: *C*. *tropicalis*				
• No	46 (63.9)	17 (81.0)	Reference	
• Yes	26 (36.1)	4 (19.0)	0.42 (0.11, 1.27)	0.149
Candida species: *C*. *glabrata*				
• No	56 (77.8)	14 (66.7)	Reference	
• Yes	16 (22.2)	7 (33.3)	1.75 (0.58, 5.00)	0.303
Candida species: *C*. *parapsilosis*				
• No	66 (91.7)	19 (90.5)	Reference	
• Yes	6 (8.3)	2 (9.5)	1.16 (0.16, 5.51)	0.864
ICU stay				
• No	36 (50.0)	9 (42.9)	Reference	
• Yes	36 (50.0)	12 (57.1)	1.33 (0.50, 3.64)	0.565
Ongoing TPN use				
• No	61 (84.7)	18 (85.7)	Reference	
• Yes	11 (15.3)	3 (14.3)	0.92 (0.19, 3.35)	0.911
Presence of central line				
• No	14 (19.4)	6 (28.6)	Reference	
• Yes	58 (80.6)	15 (71.4)	0.60 (0.20, 1.94)	0.373
Dialysis within last 3 days				
• No	45 (62.5)	15 (71.4)	Reference	
• Yes	27 (27.5)	6 (28.6)	0.67 (0.22, 1.86)	0.454
Broad spectrum antibiotics within last 1 week^ǂ^				
• No	2 (2.8)	0 (0)	Reference	
• Yes	70 (97.2)	21 (100)	-	-
Abdominal surgery within last 1 week				
• No	63 (87.5)	18 (85.7)	Reference	
• Yes	9 (12.5)	3 (14.3)	1.17 (0.24, 4.40)	0.830
Ongoing pancreatitis				
• No	67 (93.1)	20 (95.2)	Reference	
• Yes	5 (6.9)	1 (4.8)	0.67 (0.03, 4.48)	0.722
Neutropenia (ANC<500/μL)				
• No	61 (84.7)	19 (90.5)	Reference	
• Yes	11 (15.3)	2 (9.5)	0.58 (0.09, 2.43)	0.508
Prolonged neutropenia >10 days				
• No	66 (91.7)	21 (100)	Reference	
• Yes	6 (8.3)	0 (0)	-	-
Transplant recipient				
• No	65 (90.3)	19 (90.5)	Reference	
• Yes	7 (9.7)	2 (9.5)	0.98 (0.14, 4.46)	0.978
Transplant type: allogenic HSCT				
• No	70 (97.2)	21 (100)	Reference	
• Yes	2 (2.8)	0 (0)	-	-
Transplant type: liver transplant				
• No	70 (97.2)	20 (95.2)	Reference	
• Yes	2 (2.8)	1 (4.8)	1.75 (0.08, 19.19)	0.655
Transplant type: heart or lung transplant				
• No	71 (98.6)	21 (100)	Reference	
• Yes	1 (1.4)	0 (0)	-	-
Transplant type: renal transplant				
• No	70 (97.2)	20 (95.2)	Reference	
• Yes	2 (2.8)	1 (4.8)	1.75 (0.08, 19.19)	0.655
Received prolonged at least moderate dose steroids^§^				
• No	68 (94.4)	18 (85.7)	Reference	
• Yes	4 (5.6)	3 (14.3)	2.83 (0.52, 14.00)	0.198
Use of T cell suppression within 90 days^¥^				
• No	55 (76.4)	16 (76.2)	Reference	
• Yes	17 (23.6)	5 (23.8)	1.01 (0.30, 3.03)	0.985
Underlying HIV				
• No	70 (97.2)	21 (100)	Reference	
• Yes	2 (2.8)	0 (0)	-	-
Underlying malignancy				
• No	41 (56.9)	12 (57.1)	Reference	
• Yes	31 (43.1)	9 (42.9)	0.99 (0.36, 2.64)	0.987
Malignancy type: solid organ malignancy				
• No	54 (75.0)	14 (66.7)	Reference	
• Yes	18 (26.0)	7 (33.3)	1.50 (0.50, 4.23)	0.450
Malignancy type: hematological malignancy				
• No	61 (84.7)	19 (90.4)	Reference	
• Yes	11 (15.3)	2 (9.6)	0.58 (0.09, 2.43)	0.508
Underlying chronic liver disease				
• No	66 (91.7)	20 (95.2)	Reference	
• Yes	6 (8.3)	1 (4.8)	0.55 (0.03, 3.49)	0.590
Underlying autoimmune disease				
• No	67 (93.1)	19 (90.4)	Reference	
• Yes	5 (6.9)	2 (9.6)	1.41 (0.19, 7.14)	0.695
Use of azole within 1 week				
• No	65 (90.3)	19 (90.4)	Reference	
• Yes	7 (9.7)	2 (9.6)	0.98 (0.14, 4.46)	0.978
Use of echinocandin within 1 week				
• No	67 (93.1)	19 (90.4)	Reference	
• Yes	5 (6.9)	2 (9.6)	1.41 (0.19, 7.14)	0.695
Fulfils MSG rule [13]				
• No	63 (87.5)	20 (95.2)	Reference	
• Yes	9 (12.5)	1 (4.8)	0.35 (0.02, 2.04)	0.333
Death within 30 days of positive culture				
• No	34 (47.2)	11 (52.4)	Reference	
• Yes	38 (52.8)	10 (47.6)	0.81 (0.30, 2.16)	0.678
Fungal growth detection within 48h				
• No	19 (26.4)	11 (52.4)	Reference	
• Yes	53 (73.6)	10 (47.6)	**0.33 (0.12, 0.89)**	**0.029**
Administration of appropriate anti-fungal therapy within 12 hours				
• No	39 (54.2)	13 (61.9)	Reference	
• Yes	33 (45.8)	8 (38.1)	0.73 (0.26, 1.94)	0.531
Administration of appropriate anti-fungal therapy within 24 hours				
• No	32 (44.4)	13 (61.9)	Reference	
• Yes	40 (55.6)	8 (38.1)	0.49 (0.18, 1.31)	0.163
Administration of appropriate anti-fungal therapy within 48 hours				
• No	16 (22.2)	13 (61.9)	Reference	
• Yes	56 (77.8)	8 (38.1)	**0.18 (0.06, 0.49)**	**0.001**

Abbreviations: ICU = intensive care unit; TPN = total parenteral nutrition; ANC = absolute neutrophil count; HSCT = hematopoietic stem cell transplant; HIV = human immunodeficiency virus.

ƚRefer to the Methods section for the calculation of p values

ǂBroad-spectrum antibiotics include 3rd, 4th, and 5th generation cephalosporins, amoxicillin-clavulanate, anti-pseudomonal penicillins, carbapenems, aminoglycosides, fluoroquinolones, aztreonam, tigecycline

§Mean minimum dose of 0.3 mg/kg/day of prednisolone equivalent for 13 weeks

¥Cyclosporine, TNF-α blockers, specific monoclonal antibodies, or nucleoside analogues

### Analysis C

In 40 patients with concordant blood culture sets (Table 5), the use of Myco culture bottles detected fungal growth earlier by at least one day in 11 cases (27.5%) compared to the Aer/Anaer culture bottles. In the same vein, Myco culture bottles allowed fungal speciation earlier by at least one day in 10 cases (25.0%).

**Table 5:** Time to positivity of fungal growth and time to speciation of fungus among 40 patients with concordant blood culture sets.

Positive bottle	Mean time to event (days)		No. of bottles with earlier fungal detection by ≥1 day (%)	No. of bottles with earlier fungal speciation by ≥1 day (%)
	Positive fungal growth	Fungal speciation		
Aer/Anaer bottle	2.00*	5.08	6 (15.0%)	6 (15.0%)
Myco bottle	1.83	5.00	11 (27.5)	10 (25.0)

*1 data set missing

The additional use of Myco culture bottles did not make a difference in terms of earlier detection or speciation in cases of trichosporonemia where the culture bottles are concordant (2 patients; 5 *Trichosporon asahii* isolates).

## Discussion

To date, our study is the largest series in terms of patient and fungemic episodes analysed. Among patients who had Myco bottles sent contemporaneously with Aer/Anaer culture bottles, we found that the practice of sending Myco culture bottles would have diagnosed an additional 22.6% of fungemias. In addition, there was earlier detection and speciation of fungal growth in 27.5% and 25.0% of the cases respectively.

The high yield of the dedicated fungal culture bottles is surprising as the existing data are mixed.

Horvath et al. performed a simulated candidemia experiment by inoculating standardized quantities of candida into blood culture bottles that had been inoculated with blood from healthy volunteers [14]. The bottles they tested included the Aer, Anaer, and Myco culture bottles. Without the Myco culture bottle, 3 out of 50 Candida isolates (1 *C*. *krusei*, 2 *C*. *glabrata*) were missed, based on the study’s incubation period of 12 days. If a standard 5-day incubation period had been employed, 1 additional episode of *C*. *glabrata* candidemia would have been missed, resulting in an overall 8% miss rate.

Based on Vetter’s study [4], if an Aer culture bottle was sent without a Myco culture bottle, 7 out of 22 (31.8%) fungemic isolates would have been missed. This would be consistent with our reported rate of 22.6%, given that the inclusion of the Anaer culture bottle in our study did not make a difference to the overall diagnostic yield. Fuller et al. estimated that an even higher percentage of fungemic episodes would be missed if a dedicated blood culture bottle is not used, albeit based on a smaller total number of candidemias compared to our study [15].

Kirby et al. reported that a small number of Candida isolates grew only in Myco culture bottles [5]. However, a closer look at their figures suggests that their results were not too different from ours. There were 85 episodes of candidemia in their study, but 53 of these could not be analysed as they came from non-contemporaneously submitted Aer, Anaer and Myco blood culture bottles. Of the evaluable 32 cases, 4 (12.5%) grew in the Myco culture bottles alone. This difference was not statistically significant. However, as candidemia carries much morbidity and mortality, missing 12.5% of the candidemias seems to represent a loss.

A recent abstract by Fernandez et al. concluded, through a 10-year retrospective multicentre study, that fungal blood cultures had little impact on clinical care. Yet in this study, 22 out of 64 (34.4%) unique fungal infection episodes would have been missed without the use of Myco culture bottles [16]. They had considered Aer/Anaer culture bottles to be a contemporaneous set if the bottles were sent within seven days of the Myco bottle–this could potentially underestimate the yield of the Myco culture bottle given that episodes of fungaemias may be intermittent and transient, confounding the comparison of the culture bottles when taken up to seven days apart.

Overall, our findings on the additional yield of a dedicated fungal culture bottle is not at variance with the literature. Most of the studies, while acknowledging the missed fungemic episodes without fungal bottle use, concluded that a dedicated fungal bottle use is not cost-effective. Kirby et al. even went on to embark on a hospital-wide campaign to reduce the use of fungal culture bottles.

Our study isolates consisted predominantly of *Candida* spp., which are generally regarded as obligate aerobes [17]. This suggests that Candida should grow well in the aerobic medium. However, Candida invades the bloodstream and are phagocytised by neutrophils [18]. The lysing agent in the Myco culture bottle should theoretically permit the release of phagocytised Candida. From a pathogenesis viewpoint, a dedicated fungal culture medium, or those employing lysis centrifugation, should be better than the standard aerobic/anaerobic blood culture media. Our study lends support to this theory.

In addition, our study demonstrated both earlier detection and speciation of fungal growth in about a quarter of the cases where Myco culture bottles were positive with one or more of Aer/Anaer bottles. Early commencement of anti-fungal therapy for fungemia directly affects patient mortality. Even a day’s difference is of significance. In a study of 230 patients with candidemia, the number of days that passed from notification of the first positive culture to the initiation of fluconazole correlated with mortality rates in an incremental manner: day 0 (15 percent); day 1 (24 percent); day 2 (37 percent); day 3 (41 percent) [19]. Newer technologies (eg, T2 Candida) [20] may be associated with earlier detection of fungemia, but culture allows exact speciation and an isolate for susceptibility testing. The benefit of adding a Myco culture bottle in the current practice context is clear.

An interesting finding of this study is that, in patients in whom appropriate anti-fungal therapy was initiated within 48 hours, Myco culture bottles were less useful. This finding could suggest that standard Aer/Anaer culture bottles have good yield in these cases. These patients likely have a high Candida burden with advanced disease and the early initiation of anti-fungal therapy was a straightforward decision. On the other hand, fungal culture bottles were more useful in detecting fungemia when the clinical suspicion was enough to trigger a specific order for a fungal blood culture, but not enough for the clinician to commence early empiric anti-fungal therapy. We hypothesise that this clinical picture reflected a lower burden of candidemia and the lysing agent in the Myco culture bottle would have proved beneficial [9].

The clinical diagnosis of candidemia is difficult–there are no specific signs of candidemia. One of the current debates in literature is to identify the best triggers for initiating treatment [21]. In the ICU, the use of scores to trigger anti-candida therapy has been popular, with the Candida score among the most widely-quoted [13, 22–24]. Outside the ICU, the literature provides little guidance apart from that in febrile neutropenia. However, patients from general medical wards constitute a large proportion of candidemia cases [25–28]. In these settings, the decision to commence anti-fungal therapy is almost an art. Hence, we suspect that in a study like ours, early initiation of anti-fungal therapy likely occurred in patients with a high Candida score or whose clinical circumstances simply demanded anti-fungal therapy. This may have reduced the impact of Myco culture bottles.

The manufacturer recommends that 8–10ml of blood is needed for each Aer and Anaer culture bottle [8] while only 3–5ml of blood is needed for a Myco culture bottle [9]. In our study, each contemporaneous blood culture set generally consisted of up to three pairs of Aer/Anaer culture bottles and one (or occasionally two or three) Myco culture bottle(s). Despite being a lone bottle with a smaller volume of blood needed in most instances, Myco culture bottles proved to be invaluable. Additionally, a Myco culture bottle can pick up bacteremias that are missed by Aer/Anaer culture bottles, as well as mycobacteremias, if incubated long enough (data not shown).

Here in our study population, we have a unique collection of Trichosporon cases, not seen in other studies. The Aer/Anaer pair was superior in the identification of *Trichosporon asahii*. In 4 patient episodes (with 8 positive sets sent), all isolates were picked up by the Aer/Anaer culture bottles, but not always from the Myco culture bottles. Where *Trichosporon asahii* was cultured from concordant sets, the Aer/Anaer culture bottles also yielded an earlier time to fungal detection and speciation.

Although our study represents the largest cohort till date, it has several important limitations. Firstly, as it was performed at a single centre, it may be difficult to apply our results to other settings. Secondly, as this is a retrospective study, there may be unknown biases. Thirdly, as our lab does not routinely collect data on the volume of blood inoculated into each culture bottle, a variation in the sample volume may have had an impact on our results. Fourthly, we regret that we do not have the scope for a cost-effective analysis.

## Conclusion

Our study highlights the important value of a dedicated fungal blood culture when fungemia is clinically suspected. Nearly a quarter of fungemias may be missed if a fungal blood culture is not performed.
